# Confounding Effects of Lifestyle Factors in Cancer Risk Estimation for Occupational Radiation Exposure

**DOI:** 10.1016/j.shaw.2025.06.003

**Published:** 2025-06-28

**Authors:** Eun Jung Park, Ye Jin Bang, Won Jin Lee

**Affiliations:** 1 Department of Preventive Medicine, Korea University College of Medicine, Seoul, Republic of Korea; 2 Graduate School of Public Health, Korea University, Seoul, Republic of Korea

**Keywords:** bias, cohort, healthcare workers, ionizing radiation, occupational exposure

## Abstract

**Background:**

The confounding effect of lifestyle factors is an important concern in occupational studies, particularly when the risk magnitude is relatively small. This study aimed to evaluate the potential confounding effects of lifestyle factors on the association between radiation exposure and cancer incidence.

**Methods:**

Data from all Republic of Korean diagnostic medical radiation workers enrolled in the national dose registry were merged with cancer incidence records up to 2018. Excess relative risks (ERRs) for cancer were calculated using Poisson regression models to quantify the radiation dose-response relationship. Major lifestyle factors were imputed using multiple imputations by chained equations based on survey data. The confounding effects were assessed by comparing ERRs before and after adjustment for lifestyle factors.

**Results:**

The baseline ERR for cancer incidence per Sievert was 0.44 (95% CI: -0.94, 1.83) after adjusting for attained age, sex, birth year, and employment duration. Further adjustment for lifestyle factors (smoking status, alcohol consumption, body mass index, physical exercise, sleep duration, and night shift work) did not substantially modify this risk coefficient, with change-in-estimate values ranging from 0% to 13.6%. Sensitivity analyses conducted with the survey-based cohort and sex-stratified analyses yielded consistent results.

**Conclusion:**

Our study found little evidence of significant confounding effects from unmeasured lifestyle factors on cancer risk when basic registry data variables were adjusted among medical radiation workers. Further studies are warranted to investigate the impact of unmeasured and unknown confounders to improve the accuracy of radiation risk estimates.

## 1 Introduction

Inadequate adjustment for confounders can compromise the validity of estimated associations between exposure and health outcomes, potentially leading to overestimation (positive confounding) or underestimation (negative confounding) of the true relationship [1]. In studies of low-dose radiation, controlling for confounding is particularly important because the effects of radiation are relatively small, and inadequate adjustment can distort findings and lead to incorrect conclusions [2]. Consequently, studies of cancer risk associated with low-dose radiation require a more detailed epidemiological approach to properly address potential confounders. However, studies of radiation workers are typically conducted as retrospective cohort studies, which often lack detailed information on lifestyle factors. As a result, most studies on radiation workers adjust only for basic demographic factors such as attained age, sex, and birth year, facing substantial challenges in accounting for lifestyle-related variables.

Recent systematic reviews have reported that the confounding effects of lifestyle factors on cancer risk are limited in low-dose radiation studies [3]. However, there is significant heterogeneity in the magnitude and direction of confounding effects across different study populations. For instance, smoking was identified as a significant positive confounder in the association between occupational radiation exposure and cancer mortality in Japanese nuclear workers [4], whereas it was a negative confounder in U.S. nuclear workers [5]. In addition, previous studies on cancer risk from occupational radiation exposure have primarily focused on individual confounding variables, such as smoking [[6], [7], [8], [9], [10], [11], [12]], alcohol consumption [8,9], or socioeconomic status [13]. Only a few studies, notably those from the U.S. Radiologic Technologists cohort [[14], [15], [16]] and Chinese medical X-ray workers [9], have investigated multiple lifestyle factors simultaneously. Furthermore, despite the growing proportion of female radiation workers worldwide, studies specifically examining female workers remain limited, and most previous studies have relied on mortality or cancer incidence data without integrating detailed lifestyle information.

Therefore, the objective of this study was to assess the potential confounding effects of lifestyle factors on the association between radiation exposure and cancer risk. In the Republic of Korea, we established a registry-based cohort by linking data from the National Dose Registry (NDR) with national mortality and cancer incidence records. Previous studies using this cohort have evaluated the influence of occupational radiation exposure on the incidence of all cancers [17] and the risk of circulatory disease [18]. Given the relatively low risk posed by low-dose radiation exposure, understanding its impact on potential confounding by lifestyle factors is essential when assessing health risks in radiation workers. Addressing this issue is particularly important, as large-scale registry-based occupational cohorts often lack detailed lifestyle information, and our findings may provide valuable insights for registry-based research.

## 2 Materials and methods

### 2.1 Study population

The study population and methods have been described in detail previously [17]. Briefly, the study cohort consists of 94,379 diagnostic medical radiation workers registered in the NDR of the Republic of Korea Disease Control and Prevention Agency (KDCA) from January 1, 1996, to December 31, 2011. The cohort included radiologic technologists, radiologists, physicians (nonradiologists), dentists, dental hygienists, nurses, and other medical professionals such as medical assistants. Among these workers, 5,446 participated in a self-administered questionnaire survey, which included information on lifestyle factors, conducted in 2012–2013 [19]. For nonsurvey participants, we conducted micro-level data integration of NDR and survey data. We used multiple imputations by chained equations (MICE) algorithm to impute major lifestyle factors based on observed data from survey participants, incorporating variables such as attained age, sex, job title, and facility type as predictors. In this study, we assumed that missing values were missing at random. MICE is based on a variable-by-variable basis and generates multiple imputed datasets through an iterative procedure [20].

After excluding workers diagnosed with cancer before enrollment and those with invalid data (n = 419), we constructed a registry-based cohort (n = 81,054), which included workers with imputed lifestyle data. In this analysis, we used the registry-based cohort for the primary analysis due to its larger size and greater statistical power. The survey-based cohort, which has detailed lifestyle factors (n = 5,446), was used in sensitivity analyses to assess potential biases or differences arising from directly collected lifestyle data due to the limited number of cancer cases.

The registry-based cohort did not involve direct contact with workers, and informed consent was not required. Participants in the survey-based cohort provided written informed consent, including permission for the use of radiation dosimetry data, prior to enrollment. This study was approved by the Institutional Review Board of Republic of Korea University (KUIRB-2019-0092-09).

### 2.2 Dosimetry data

Occupational exposure data were obtained from the NDR database. The NDR is a government-operated centralized dosimetry registry for all diagnostic radiation workers in the Republic of Korea, maintained by the KDCA since 1996 (https://www.kdca.go.kr). The registry information for workers' includs name, gender, personal identification number, job classification, quarterly dose data, and the beginning and end of the period of measurement. Dose measurements were collected quarterly using a personal thermoluminescence dosemeter. For workers employed before 1996, historical doses were reconstructed using a log-linear dose model that accounted for calendar year and age at exposure to estimate missing doses [21].

### 2.3 Ascertainment of cancer incidence and vital status

Cancer incidence was identified through linkage with the Korean Central Cancer Registry (KCCR), a national cancer registry maintained by the Korean National Cancer Center. The KCCR provides detailed information on cancer diagnoses, including cancer sites, histological types, stages, and diagnosis dates. Vital status was confirmed through linkage with Statistics Republic of Korea (http://kostat.go.kr) using a similar method. Solid cancers were defined as the first primary malignant tumors classified by the International Classification of Diseases, 10th Revision (ICD-10) codes C00–C80. Cancer incidence was followed up until 31 December 2018. The nationwide completeness of cancer data in the Republic of Korea is estimated to be 98.2% [22].

### 2.4 Identification of potential confounders

**Fig. 1:**
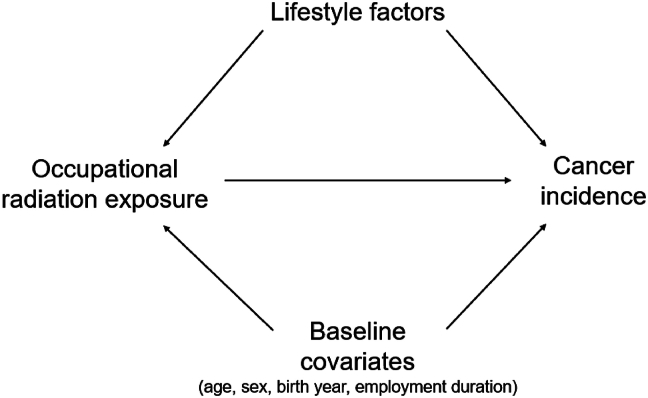
Directed acyclic graph describing the relationship between occupational radiation exposure and cancer incidence. The baseline model included attained age, sex, birth year, and employment duration. Selected potential confounders are smoking status, alcohol consumption, BMI, physical exercise, sleep duration, night shift work. BMI, body mass index.

A directed acyclic graph was constructed to identify potential lifestyle factors that could confound the association between occupational radiation exposure and solid cancer (Fig. 1). In this directed acyclic graph, confounders were categorized into baseline factors (attained age, sex, birth year, and employment duration), typically applied in registry-based cohorts, and lifestyle-related factors that may influence both radiation exposure and cancer risk, with directed arrows indicating the assumed causal relationships. Selected lifestyle factors were smoking status, alcohol consumption, body mass index (BMI; calculated as weight in kilograms divided by height in meters squared), physical exercise, sleep duration, and night shift work. These factors were chosen based on prior literature on cancer risk (https://monographs.iarc.who.int/) and their practical relevance as occupational and lifestyle characteristics of the study population, which are commonly encountered by healthcare professionals.

Smoking status was defined as never or ever, based on a lifetime history of smoking at least 100 cigarettes. Alcohol consumption was classified as never or ever, based on current drinking status. Physical exercise was classified as never or ever, based on engagement in regular activity intense enough to cause sweating. BMI was classified as <23.0 or ≥23.0 kg/m^2^, based on values calculated using self-reported height and weight. Sleep duration was classified as <7 or ≥7 hours, based on average daily sleep. Night shift work was classified as never or ever, based on working night shifts at least three times per month.

### 2.5 Data analysis

Each worker contributed person-years from the start of their work with radiation (1996 or the first year of work, whichever was later) until the date of solid cancer diagnosis, death, or December 31, 2018. The DATAB module of Epicure software was used to create a person-year table stratified by sex, attained age (<25 years, 5-year intervals from 25–84, ≥85 years), calendar year (1996–2000, 2001–2005, 2006–2010, 2011–2018), birth year (<1960, 1960–1969, 1970–1979, ≥1980), job title (physician, nonphysician), age at first job (<25, 25–30, 31–35, 36–40, ≥40), job start year (<1996, 1996–2004, ≥2005), employment duration (<1, 1–4, 5–9, ≥10 years), type of medical facility (general hospital, hospital and clinic, dental hospital and clinic, others), geographic location (metropolitan, city, rural), cumulative badge dose (<1, 1–4, 5–19, ≥20 mSv), smoking status (never, ever), alcohol consumption (never, ever), BMI (<23.0, ≥23.0 kg/m^2^), physical exercise (never, ever), sleep duration (<7, ≥7 hours/day), and night shift work (never, ever).

Excess relative risks (ERRs) and 95% confidence intervals (CIs) for cancer incidence were estimated using Poisson regression to analyze the relationship between cumulative badge doses (5-year lag) and solid cancer incidence. The primary model assumed a linear dose-response relationship, commonly applied in radiation epidemiology [2]. The model can be expressed as: RR = 1 + βd, where RR is the relative risk, d is the dose, and β is an estimate of the excess relative risk per unit dose (ERR/Sv). Parameter estimates and 95% CIs were obtained using the maximum likelihood method. Model selection was guided by deviance and the Akaike information criterion (AIC). The final baseline models were adjusted for attained age, sex, birth year, and employment duration, consistent with previous analyses [17]. All statistical analyses were conducted using the AMFIT module in Epicure (Risk Sciences International, version 2.0; Ottawa, Canada).

Mann–Whitney U tests were used to assess the association between lifestyle factors and cumulative badge dose, while chi-squared tests were conducted to analyze associations between lifestyle factors and solid cancer incidence. The potential effects of confounding were assessed by comparing the ERR estimates before and after adjustment for lifestyle factors. The percentage change in the ERR was used to quantify the magnitude of confounding. Specifically, the change-in-estimate was calculated as the proportional difference between the baseline ERR (adjusted only for attained age, sex, birth year, and employment duration) and the ERR after additional including lifestyle factors in the model. The presence of a confounding effect was defined as a change-in-estimate of 20% or more before and after adjustment. Stratified analyses were performed to assess potential effect modification by levels of confounders. Sensitivity analyses were conducted using the survey-based cohort (n = 5,446) to explore differences in confounding effects compared with the registry-based cohort. Additionally, analyses were stratified by sex to account for differences in baseline rates.

## 3 Results

Among the 81,054 cohort members (44,625 men and 36,429 women), a total of 3,243 solid cancer cases were identified, comprising 1,944 cases in men and 1,299 cases in women (Table 1). The majority of workers were born after 1960, and approximately half started work after 2004. The median attained age at the end of the follow-up was 39.0 years. Most workers (81.0%) had been employed for less than 10 years. The mean cumulative badge dose was 5.7 mSv (0.005 mSv–603.6 mSv), and the dose distribution was highly skewed, with 56.2 % of all workers having cumulative badge doses below 1 mSv. The survey-based cohort consisted predominantly of radiologic technologists, who had higher radiation doses compared to other occupational groups (Supplementary Table 1).

**Table 1:** Occupational characteristics of Republic of Korean diagnostic medical radiation workers, stratified by solid cancer status, 1996-2018.

Characteristics	Total		Cases		Noncases	
	Number (%)		Number (%)		Number (%)	
Total	81,054	(100.0)	3,243	(100.0)	77,811	(100.0)
Sex						
Male	44,625	(55.1)	1,944	(59.9)	42,681	(54.8)
Female	36,429	(44.9)	1,299	(40.1)	35,130	(45.2)
Occupation						
Physician	34,632	(42.7)	1,716	(52.9)	32,916	(42.3)
Nonphysician	46,422	(57.3)	1,527	(47.1)	44,895	(57.7)
Type of facility						
General hospital	16,011	(19.7)	630	(19.4)	15,381	(19.8)
Hospital and clinic	30,279	(37.4)	1,447	(44.6)	28,832	(37.1)
Dental hospital and clinic	31,061	(38.3)	1,007	(31.1)	30,054	(38.6)
Others	3,703	(4.6)	159	(4.9)	3,544	(4.5)
Area of facility						
Metropolitan	43,330	(53.5)	1,829	(56.4)	41,501	(53.4)
City	32,678	(40.3)	1,215	(37.5)	31,463	(40.4)
Rural	5,046	(6.2)	199	(6.1)	4,847	(6.2)
Calendar year of birth						
<1960	9,751	(12.0)	1,193	(36.8)	8,558	(11.0)
1960–1969	21,732	(26.8)	952	(29.4)	20,780	(26.7)
1970–1979	28,514	(35.2)	809	(24.9)	27,705	(35.6)
≥1980	21,057	(26.0)	289	(8.9)	20,768	(26.7)
Age at entry (years)						
<25	23,961	(29.5)	725	(22.3)	23,236	(29.9)
25–29	21,051	(26.0)	706	(21.8)	20,345	(26.1)
30–34	13,261	(16.4)	494	(15.2)	12,767	(16.4)
35–39	11,046	(13.6)	466	(14.4)	10,580	(13.6)
≥40	11,735	(14.5)	852	(26.3)	10,883	(14.0)
Calendar year of work began						
<1996	10,397	(12.8)	893	(27.5)	9,504	(12.2)
1996–2004	30,848	(38.1)	1,362	(42.0)	29,486	(37.9)
≥2005	39,809	(49.1)	988	(30.5)	38,821	(49.9)
Duration of employment (years)						
<1	16,978	(21.0)	446	(13.7)	16,532	(21.2)
1–4	29,497	(36.4)	930	(28.7)	28,567	(36.7)
5–9	19,149	(23.6)	729	(22.5)	18,420	(23.7)
≥10	15,430	(19.0)	1,138	(35.1)	14,292	(18.4)
Cumulative badge dose (mSv)						
<1	45,583	(56.2)	1,441	(44.4)	44,142	(56.7)
1–4	18,357	(22.7)	744	(22.9)	17,613	(22.6)
5–19	11,094	(13.7)	550	(17.0)	10,544	(13.6)
≥20	6,020	(7.4)	508	(16.7)	5,512	(7.1)

Table 2 shows the crude associations between lifestyle factors, cumulative badge dose, and solid cancers. Workers with statistically higher occupational radiation doses were more likely to be smokers, nondrinkers, have a BMI of 23.0 kg/m^2^ or higher, engage in regular exercise, sleep less, and work shifts, compared to their counterparts. Solid cancer cases were more common among ever-smokers, ever-drinkers, those with a BMI of less than 23.0 kg/m^2^, and those who exercised regularly but did not work shifts. Similar patterns were observed for both male and female workers (Supplementary Table 2).

**Table 2:** Association of lifestyle factors with cumulative badge dose and solid cancer in the registry-based cohort of Republic of Korean diagnostic medical radiation workers, 1996-2018.

Lifestyle factors	Cumulate badge dose (mSv)		*p*-value∗	Cases		Noncases		*p*-value∗
	Number of workers	Mean ± SD		Number	(%)	Number	(%)	
Total	81,054	5.7 ± 17.1		3,243	(4.0)	77,811	(96.0)	
Smoking status			<0.001					<0.001
Never	44,012	4.1 ± 12.8		1,365	(42.1)	42,647	(54.8)	
Ever	37,042	7.7 ± 21.0		1,878	(57.9)	35,164	(45.2)	
Alcohol consumption (per month)			<0.001					<0.001
Never	24,779	6.1 ± 19.9		1,262	(38.9)	23,517	(30.2)	
Ever	56,275	5.6 ± 15.8		1,981	(61.1)	54,294	(69.8)	
BMI (kg/m^2^)			<0.001					<0.001
<23.0	47,267	4.1 ± 13.1		1,678	(51.7)	45,589	(58.6)	
≥23.0	33,787	8.0 ± 21.4		1,565	(48.3)	32,222	(41.4)	
Physical exercise (days per week)			<0.001					<0.001
Never	41,635	4.8 ± 14.4		1,368	(42.2)	40,267	(51.8)	
Ever	39,419	6.7 ± 19.5		1,875	(57.8)	37,544	(48.3)	
Sleep duration (hour per day)			<0.001					0.324
<7	27,665	5.8 ± 17.9		1,133	(34.9)	26,532	(34.1)	
≥7	53,389	5.7 ± 16.7		2,110	(65.1)	51,279	(65.9)	
Night shift work			<0.001					0.020
Never	56,311	4.5 ± 15.2		2,313	(71.3)	53,998	(69.4)	
Ever	24,743	8.5 ± 20.5		930	(28.7)	23,813	(30.6)	

BMI, body mass index; SD, standard deviation.

∗ *P*-values are based on the Mann-Whitney U Test or chi-squared test.

Table 3 presents the ERRs for solid cancer and the change-in-estimate after adjusting for potential confounders. The baseline ERR per Sv for solid cancer with a 5-year lag was 0.44 (95% CI: -0.94, 1.83) after adjustment for attained age, sex, birth year, and employment duration. Adjustments for individual lifestyle factors resulted in minor changes in the ERR estimates: smoking status (2.2%), alcohol consumption (4.5%), BMI (0.0%), physical exercise (4.5%), sleep duration (0.0%), and night shift work (13.6%). None of these changes were statistically significant compared to the baseline model. Similar patterns were observed for both male and female workers, although the magnitude of ERRs differed by sex. This pattern was consistent in the survey-based cohort (Supplementary Table 3).

**Table 3:** Excess relative risk per Sv and change-in-estimate for lifestyle factors in the registry-based cohort of Republic of Korean diagnostic medical radiation workers, 1996-2018.

Models	Total			Male			Female		
	ERR/Sv (95% CI)	CIE (%)	AIC	ERR/Sv (95% CI)	CIE (%)	AIC	ERR/Sv (95% CI)	CIE (%)	AIC
Baseline model∗	0.44 (-0.94, 1.83)	-	32750.0	0.59 (-0.89, 2.07)	-	19416.8	-1.41 (-5.62, 2.80)	-	13240.4
+ Smoking status	0.45 (-0.94, 1.83)	-2.2	32750.7	0.60 (-0.89, 2.08)	-1.7	19418.2	-1.47 (-5.56, 2.61)	-4.3	13241.0
+ Alcohol consumption	0.46 (-0.94, 1.85)	-4.5	32750.8	0.61 (-0.88, 2.10)	-3.4	19417.4	-1.41 (-5.62, 2.80)	0.0	13242.3
+ BMI	0.44 (-0.94, 1.83)	0.0	32751.5	0.59 (-0.89, 2.07)	0.0	19418.4	-1.40 (-5.62, 2.82)	0.7	13242.2
+ Physical exercise	0.46 (-0.93, 1.85)	-4.5	32749.5	0.61 (-0.87, 2.09)	-3.4	19417.7	-1.42 (-5.59, 2.75)	-0.7	13240.8
+ Sleep duration	0.44 (-0.95, 1.82)	0.0	32751.0	0.59 (-0.89, 2.07)	0.0	19418.8	-1.60 (-5.42, 2.22)	-13.5	13239.3
+ Night shift work	0.50 (-0.90, 1.90)	-13.6	32749.6	0.70 (-0.80, 2.21)	-18.6	19411.3	-1.57 (-5.53, 2.40)	-11.3	13241.4

AIC, Akaike information criteria; BMI, body mass index; CI, confidence interval; CIE, change-in-estimate; ERR, excess relative risk.

∗ Adjusted for sex, attained age (<25, 5-year intervals from the age of 25–84, ≥85 years), birth year (<1960, 1960–1969, 1970–1979, ≥1980) and years of employment duration (<1, 1–4, 5–9, ≥10).

The potential for effect modification was assessed by comparing stratum-specific estimates (Table 4). Although some heterogeneity across strata was observed, the differences were not statistically significant as the 95% CIs largely overlapped. These findings were consistent between male and female workers.

**Table 4:** Excess relative risk stratified by lifestyle factors in the registry-based cohort of Republic of Korean diagnostic medical radiation workers, 1996-2018.

Lifestyle factors	Total		Male		Female	
	Cases	ERR/Sv (95% CI)	Cases	ERR/Sv (95% CI)	Cases	ERR/Sv (95% CI)
Smoking status						
Never	1,365	2.56 (-1.10, 6.21)	594	1.49 (-1.91, 4.89)	771	12.10 (-5.67, 29.86)
Ever	1,878	0.03 (-1.46, 1.52)	1,350	0.43 (-1.24, 2.10)	528	-1.95 (-3.90, -0.01)
Alcohol consumption (per month)						
Never	1,262	-0.67 (-2.16, 0.83)	688	-0.68 (-2.22, 0.85)	574	-2.17 (-2.46, -1.87)
Ever	1,981	1.87 (-0.60, 4.35)	1,256	2.14 (-0.52, 4.79)	725	1.56 (-8.71, 11.82)
BMI (kg/m^2^)						
<23.0	1,678	-0.50 (-2.94, 1.94)	560	0.01 (-3.00, 3.02)	1,118	-1.44 (-5.85, 2.97)
≥23.0	1,565	0.76 (-0.93, 2.46)	1,384	0.68 (-1.00, 2.37)	181	0.74 (-14.20, 15.68)
Physical exercise (days per week)						
Never	1,368	0.71 (-2.12, 3.54)	598	0.44 (-2.40, 3.28)	770	-1.51 (-8.94, 5.93)
Ever	1,875	0.43 (-1.19, 2.04)	1346	0.68 (-1.07, 2.42)	529	-1.80 (-5.59, 1.99)
Sleep duration (hour per day)						
<7	1,133	0.79 (-1.65, 3.22)	595	1.44 (-1.42, 4.29)	538	-2.00 (-2.46, -1.54)
≥7	2,110	0.20 (-1.46, 1.86)	1349	0.17 (-1.53, 1.87)	761	1.82 (-7.89, 11.53)
Night shift work						
Never	2,313	0.22 (-1.47, 1.91)	1322	0.44 (-1.41, 2.29)	991	-0.33 (-5.83, 5.16)
Ever	930	1.12 (-1.43, 3.67)	622	1.11 (-1.48, 3.70)	308	-6.17 (-7.88, -4.47)

BMI, body mass index; CI, confidence interval; ERR, excess relative risk.

## 4 Discussion

Our findings provide little evidence of significant confounding effects from lifestyle factors on the association between occupational radiation exposure and cancer incidence among medical radiation workers. These effects were consistent between registry-based workers with imputed lifestyle information and survey-based workers with directly measured lifestyle data. In addition, male and female workers showed similar patterns. These results suggest that the impact of occupational radiation exposure on cancer incidence may not be a significant concern when baseline factors—such as attained age, sex, birth year, and employment duration—are adjusted using registry data. However, as the confounding effects of lifestyle factors may vary by study population and specific health outcomes, caution is needed in interpreting these findings. Further studies are needed to investigate the potential impact of unmeasured or unknown confounders for more precise effect estimates of radiation exposure.

The absence of significant confounding effects from lifestyle factors in our study may be attributed to adjustments for attained age, sex, and birth year in the baseline model. These adjustments may have already controlled for the effects of major lifestyle factors and minimized the confounding influence of individual factors. Notably, birth year has been identified as a possible surrogate for smoking-related risk factors in studies on U.S. radiation workers [7]. In addition, the relatively homogeneous characteristics of workers within a specific occupational group are likely to minimize uncontrolled lifestyle confounding [23]. In our cohort, medical radiation workers exhibited homogeneity as hospital workers, and no substantial disparity in the ERRs for cancer was observed between physicians and non-physicians [17].

Although crude associations between lifestyle factors, cumulative radiation dose, and cancer reached statistical significance, the magnitude of change in effect estimates was small and did not indicate substantial confounding effects in our cohort. This may be due to the small absolute differences in the distribution of lifestyle factors between low- and high-exposure groups. Additionally, the large sample size may have led to statistically significant findings without reflecting meaningful differences. This highlights the limitations of relying solely on crude statistical associations to identify potential confounders in epidemiological studies. Even if confounding was present in the crude associations, it may have been directly or indirectly accounted for through adjustment for baseline confounders. Furthermore, the confounding effects of lifestyle factors may be weaker for solid cancers compared to their influence on specific organ sites, such as the association between smoking and lung cancer. Organ-specific analyses with extended follow-up are warranted to further investigate these possibilities.

Our findings align with previous reviews suggesting that lifestyle factors do not significantly confound the association between occupational radiation exposure and cancer risk in low-dose radiation studies [3]. Smoking, one of the most significant confounders, did not exhibit a confounding effect in our cohort. This finding is consistent with the INWORKS study [11] and studies of U.S. nuclear facility workers [12] and Chinese X-ray workers [9]. However, other studies, such as the Japanese nuclear worker study, reported smoking as a positive confounder for solid cancers [4,8]. These discrepancies may stem from differences in population characteristics or variations in the relationships between exposure, confounders, and outcomes. Such findings underscore the importance of study-specific analyses in identifying potential confounders.

In addition to smoking, our study provides insights into the potential confounding effects of alcohol consumption, BMI, physical exercise, sleep duration, and night shift work. Most radiation worker studies have been unable to adjust for these factors due to a lack of detailed lifestyle information. Although the U.S. radiologic technologists cohort collected data on several lifestyle factors and adjusted for BMI [15,16] and physical exercise [14], these studies did not report changes in estimates, making it difficult to assess the magnitude of confounding. In our study, no significant confounding effects of sleep duration or night shift work were observed. Given that smoking did not exhibit substantial confounding effects, it is reasonable to assume that other lifestyle factors with weaker associations with cancer are also unlikely to demonstrate confounding effects. Although night shift work was not identified as a statistically significant confounder in this analysis, its biological plausibility and occupational relevance support the need for careful consideration of night shift work in medical radiation studies.

The findings from the registry-based cohort with imputed lifestyle data were consistent with those from the survey-based cohort with direct data. Differences in ERR magnitudes between the two cohorts might reflect chance due to sample size differences or the selection of participants in the self-reported survey cohort. Sensitivity analysis comparing models with and without imputed lifestyle data in the registry-based cohort showed minimal differences in ERR estimates, indicating that the imputation procedure had limited influence on the results (Supplementary Table 4). These results support the utility of multiple imputations in minimizing confounding when detailed lifestyle information is unavailable in registry-based studies. In addition, the observed variations of ERR estimates across subgroups may primarily be attributed to limited statistical power, a common constraint in low-dose radiation studies due to low cumulative exposure, short follow-up periods, and the relatively small number of cancer cases needed to detect health effects at low levels of radiation exposure. Therefore, the imprecise or negative ERR estimates observed in this study should not be interpreted as indicating the absence of risk or a protective effect of radiation.

Our study has several strengths. First, the use of detailed self-reported survey data allowed us to assess several potential lifestyle confounders by sex. Second, compared to previous studies, our cohort included a relatively high proportion of female workers (44.9%), enabling a more comprehensive analysis of sex-specific confounding effects. Third, unlike most previous studies that relied on mortality data, our study benefited from linkage to comprehensive national cancer incidence data, encompassing all monitored diagnostic medical radiation workers in the Republic of Korea. However, this study also has limitations. The imputed data in the registry-based cohort were not based on actual measurements, which may have resulted in residual confounding. Additionally, the relatively small proportion of survey participants compared with the registry-based cohort limits the generalizability of these findings. Furthermore, lifestyle factors were measured only once at baseline, which limited our ability to account for time-varying confounding and the latency period between exposure to confounders and cancer development. Therefore, the results should be interpreted with caution due to the potential influence of unmeasured or unknown confounders.

In conclusion, our findings provide little evidence that unmeasured lifestyle factors substantially confound the association between radiation exposure and cancer risk among medical radiation workers, after adjusting for baseline factors such as attained age, sex, birth year, and employment duration. This highlights the importance of baseline adjustments in occupational epidemiology, particularly when detailed lifestyle data are not available. While the potential for residual confounding remains, adjustment for baseline factors available in registry data can help minimize bias. However, the confounding effects of lifestyle factors are likely to vary between study populations, highlighting the need for population-specific evaluations. Further studies are warranted to assess the impact of unmeasured confounders and to quantify the bias introduced by each potential confounder.

## CRediT authorship contribution statement

**Eun Jung Park:** Writing – original draft, Visualization, Validation, Formal analysis, Data curation. **Ye Jin Bang:** Writing – review & editing, Visualization, Validation, Formal analysis, Data curation. **Won Jin Lee:** Writing – review & editing, Supervision, Methodology, Investigation, Conceptualization.

## Ethical statement

This study was reviewed and approved by the Institutional Review Board of Korea University (KUIRB-2019-0092-09).

## Author’s contributions

WJL conceived the idea for the study and its design, interpreted the results, and critically revised the manuscript. EJP and YJB are equally contributed as the first authors, EJP undertook the statistical analysis, interpretation of the results, and wrote the draft. YJB contributed to data analysis, interpretation of the results, and wrote and revised the manuscript. All authors gave final approval of the final version and agree to be accountable for all aspects of the published work.

## Data availability statements

The raw data for this study are not publicly available to preserve individuals' privacy, in accordance with informed consents and the approval of the Institutional Review Board.

## Declaration of Generative AI and AI-assisted technologies in the writing process

None declared.

## Funding

This work was supported by the National Research Foundation of Korea (NRF) grant funded by the Korean government (MSIT) (No. RS-2023-00208314).

## Conflicts of interest

The authors declare no conflict of interest.
