# Supplementary material for: Confounding Effects of Lifestyle Factors in Cancer Risk Estimation for Occupational Radiation Exposure
Source: Saf Health Work. 2025 Jun 28;16(3):310–6. doi: 10.1016/j.shaw.2025.06.003 (PMC12490584; doi:10.1016/j.shaw.2025.06.003)
Supplement: Multimedia component 1 [file mmc1.docx]

Supplementary Table 1. Occupational characteristics of the survey-based cohort of South Korean diagnostic medical radiation workers, stratified by solid cancer status, 1996–2018

| Characteristics | Total | | Cases | | Non-cases | |
| --- | --- | --- | --- | --- | --- | --- |
|  | Number | (%) | Number | (%) | Number | (%) |
| Total | 5,446 | (100.0) | 136 | (100.0) | 5,310 | (100.0) |
| Sex |  |  |  |  |  |  |
| Male | 3,562 | (65.4) | 83 | (61.0) | 3,479 | (65.5) |
| Female | 1,884 | (34.6) | 53 | (39.0) | 1,831 | (34.5) |
| Occupation |  |  |  |  |  |  |
| Physician | 797 | (14.6) | 25 | (18.4) | 772 | (14.5) |
| Non-physician | 4,649 | (85.4) | 111 | (81.6) | 4,538 | (85.5) |
| Type of facility |  |  |  |  |  |  |
| General hospital | 994 | (18.3) | 30 | (22.1) | 964 | (18.2) |
| Hospital and clinic | 3,275 | (60.1) | 80 | (58.8) | 3,195 | (60.2) |
| Dental hospital and clinic | 681 | (12.5) | 9 | (6.6) | 672 | (12.7) |
| Others | 496 | (9.1) | 17 | (12.5) | 479 | (9.0) |
| Area of facility |  |  |  |  |  |  |
| Metropolitan | 2,886 | (53.0) | 69 | (50.7) | 2,817 | (53.1) |
| City | 2,206 | (40.5) | 53 | (39.0) | 2,153 | (40.6) |
| Rural | 354 | (6.5) | 14 | (10.3) | 340 | (6.4) |
| Calendar year of birth |  |  |  |  |  |  |
| <1960 | 370 | (6.8) | 39 | (28.7) | 331 | (6.2) |
| 1960–1969 | 1,210 | (22.2) | 42 | (30.9) | 1,168 | (22.0) |
| 1970–1979 | 1,923 | (35.3) | 36 | (26.5) | 1,887 | (35.5) |
| ≥1980 | 1,943 | (35.7) | 19 | (14.0) | 1,924 | (36.2) |
| Age at entry (years) |  |  |  |  |  |  |
| <25 | 2,567 | (47.1) | 61 | (44.9) | 2,506 | (47.2) |
| 25–29 | 1,843 | (33.8) | 39 | (28.7) | 1,804 | (34.0) |
| 30–34 | 423 | (7.8) | 9 | (6.6) | 414 | (7.8) |
| 35–39 | 298 | (5.5) | 7 | (5.2) | 291 | (5.5) |
| ≥40 | 315 | (5.8) | 20 | (14.7) | 295 | (5.6) |
| Calendar year of work began |  |  |  |  |  |  |
| <1996 | 1,085 | (19.9) | 59 | (43.4) | 1,026 | (19.3) |
| 1996–2004 | 1,913 | (35.1) | 49 | (36.0) | 1,864 | (35.1) |
| ≥2005 | 2,448 | (45.0) | 28 | (20.6) | 2,420 | (45.6) |
| Duration of employment (years) |  |  |  |  |  |  |
| <1 | 491 | (9.0) | 8 | (5.9) | 483 | (9.1) |
| 1–4 | 1,448 | (26.6) | 16 | (11.8) | 1,432 | (27.0) |
| 5–9 | 1,356 | (24.9) | 31 | (22.8) | 1,325 | (25.0) |
| ≥10 | 2,151 | (39.5) | 81 | (59.6) | 2,070 | (39.0) |
| Cumulative badge dose (mSv) |  |  |  |  |  |  |
| <1 | 1,363 | (25.0) | 20 | (14.7) | 1,343 | (25.3) |
| 1–4 | 1,281 | (23.5) | 28 | (20.6) | 1,253 | (23.6) |
| 5–19 | 1,511 | (27.8) | 43 | (31.6) | 1,468 | (27.7) |
| ≥20 | 1,291 | (23.7) | 45 | (33.1) | 1,246 | (23.5) |

Supplementary Table 2. Association of lifestyle factors, cumulative badge dose, and solid cancer by sex in the registry-based cohort of South Korean diagnostic medical radiation workers, 1996-2018

| Lifestyle factors | Cumulate badge dose (mSv) | | *p*-value^*^ | Cases | | Non-cases | | *p*-value^*^ |
| --- | --- | --- | --- | --- | --- | --- | --- | --- |
|  | Number of workers | Mean ± SD |  | Number | (%) | Number | (%) |  |
| **Male** | 44,625 | 8.5 ± 21.6 |  | 1,944 | (4.4) | 42,681 | (95.6) |  |
| Smoking status |  |  | <0.001 |  |  |  |  | <0.001 |
| Never | 18,774 | 7.2 ± 18.1 |  | 594 | (30.6) | 18,180 | (42.6) |  |
| Ever | 25,851 | 9.5 ± 23.7 |  | 1,350 | (69.4) | 24,501 | (57.4) |  |
| Alcohol consumption (per month) |  |  | 0.002 |  |  |  |  | <0.001 |
| Never | 11,502 | 9.8 ± 26.7 |  | 688 | (35.4) | 10,814 | (25.3) |  |
| Ever | 33,123 | 8.1 ± 19.4 |  | 1,256 | (64.6) | 31,867 | (74.7) |  |
| BMI (kg/m^2^) |  |  | 0.329 |  |  |  |  | <0.001 |
| <23.0 | 15,361 | 8.1 ± 19.4 |  | 560 | (28.8) | 14,801 | (34.7) |  |
| ≥23.0 | 29,264 | 8.8 ± 22.6 |  | 1,384 | (71.2) | 27,880 | (65.3) |  |
| Physical exercise (days per week) |  |  | 0.007 |  |  |  |  | <0.001 |
| Never | 18,083 | 8.1 ± 19.8 |  | 598 | (30.8) | 17,485 | (41.0) |  |
| Ever | 26,542 | 8.8 ± 22.7 |  | 1,346 | (69.2) | 25,196 | (59.0) |  |
| Sleep duration (hour per day) |  |  | <0.001 |  |  |  |  | 0.121 |
| <7 | 12,962 | 9.8 ± 23.4 |  | 595 | (30.6) | 12,367 | (29.0) |  |
| ≥7 | 31,663 | 8.0 ± 20.7 |  | 1,349 | (69.4) | 30,314 | (71.0) |  |
| Night shift work |  |  | <0.001 |  |  |  |  | 0.001 |
| Never | 28,751 | 6.8 ± 19.5 |  | 1,322 | (68.0) | 27,429 | (58.4) |  |
| Ever | 15,874 | 11.7 ± 24.5 |  | 622 | (32.0) | 15,252 | (41.6) |  |
| **Female** | 36,429 | 2.3 ± 7.9 |  | 1,299 | (3.6) | 35,130 | (96.4) |  |
| Smoking status |  |  | <0.001 |  |  |  |  | <0.001 |
| Never | 25,238 | 1.8 ± 5.4 |  | 771 | (59.4) | 24,467 | (69.6) |  |
| Ever | 11,191 | 3.5 ± 11.6 |  | 528 | (40.6) | 10,663 | (30.4) |  |
| Alcohol consumption (per month) |  |  | <0.001 |  |  |  |  | <0.001 |
| Never | 13,277 | 2.8 ± 9.9 |  | 574 | (44.2) | 12,703 | (36.2) |  |
| Ever | 23,152 | 2.0 ± 6.4 |  | 725 | (55.8) | 22,427 | (63.8) |  |
| BMI (kg/m^2^) |  |  | <0.001 |  |  |  |  | 0.091 |
| <23.0 | 31,906 | 2.2 ± 7.8 |  | 1,118 | (86.1) | 30,788 | (87.6) |  |
| ≥23.0 | 4,523 | 2.8 ± 8.4 |  | 181 | (13.9) | 4,342 | (12.4) |  |
| Physical exercise (days per week) |  |  | <0.001 |  |  |  |  | <0.001 |
| Never | 23,552 | 2.2 ± 7.2 |  | 770 | (59.3) | 22,782 | (64.9) |  |
| Ever | 12,877 | 2.5 ± 9.0 |  | 529 | (40.7) | 12,348 | (35.1) |  |
| Sleep duration (hour per day) |  |  | <0.001 |  |  |  |  | 0.430 |
| <7 | 14,703 | 2.3 ± 9.5 |  | 538 | (41.4) | 14,165 | (40.3) |  |
| ≥7 | 21,726 | 2.3 ± 6.6 |  | 761 | (58.6) | 20,965 | (59.7) |  |
| Night shift work |  |  | <0.001 |  |  |  |  | 0.587 |
| Never | 27,560 | 2.1 ± 8.1 |  | 991 | (76.3) | 26,569 | (75.6) |  |
| Ever | 8,869 | 2.8 ± 7.2 |  | 308 | (23.7) | 8,561 | (24.4) |  |

BMI, body mass index; SD, standard deviation.

^*^*P*-values are based on the Mann-Whitney U Test or chi-squared test.

Supplementary Table 3. Excess relative risk per Sv and change-in-estimate for lifestyle factors in the survey-based cohort of South Korean diagnostic medical radiation workers, 1996-2018

| Models | ERR/Sv (95% CI) | CIE (%) | AIC |
| --- | --- | --- | --- |
| Baseline model^*^ | 4.28 (-5.47, 14.03) | - | 1648.3 |
| + Smoking status | 4.68 (-5.45, 14.81) | -9.3 | 1651.8 |
| + Alcohol consumption (per month) | 4.40 (-5.45, 14.24) | -2.8 | 1652.0 |
| + BMI (kg/m^2^) | 4.17 (-5.56, 13.90) | 2.6 | 1651.4 |
| + Physical exercise (days per week) | 4.19 (-5.47, 13.85) | 2.1 | 1649.1 |
| + Sleep duration (hour per day) | 4.28 (-5.45, 14.00) | 0.0 | 1650.7 |
| + Night shift work | 3.44 (-5.74, 12.62) | 19.6 | 1647.9 |
| + Personal medical examination | 3.89 (-5.59, 13.37) | 9.1 | 1651.3 |
| + Past medical history^†^ | 4.32 (-5.44, 14.08) | -0.9 | 1650.8 |

AIC, akaike information criteria; BMI, body mass index; CI, confidence interval; CIE, change-in-estimate; ERR, excess relative risk.

^*^Adjusted for sex, attained age (<25, 5-year intervals from the age of 25–84, ≥85 years), birth year (<1960, 1960–1969, 1970–1979, ≥1980) and years of employment duration (<1, 1–4, 5–9, ≥10).

^†^History of past diseases excluding cancer, including thyroid disorders, cardiovascular diseases, metabolic disorders, respiratory diseases, and infectious diseases.

Supplementary Table 4. Sensitivity analysis of ERR estimates from models with and without imputed lifestyle factors in the registry-based cohort

| Models | Covariates included | ERR/Sv (95% CI) | AIC |
| --- | --- | --- | --- |
| 1 | Without imputation (attained age, sex, birth year, work duration) | 0.44 (-0.94, 1.83) | 32750.0 |
| 2 | With imputation (attained age, sex, birth year, work duration, smoking status, alcohol consumption, BMI, physical exercise, sleep duration, night shift work) | 0.52 (-0.89, 1.93) | 32752.6 |

AIC, akaike information criteria; BMI, body mass index; CI, confidence interval; ERR, excess relative risk.
